# Suspected metastatic adrenocortical carcinoma revealing as pulmonary Kaposi sarcoma in adrenal Cushing’s syndrome

**DOI:** 10.1186/1472-6823-14-63

**Published:** 2014-07-30

**Authors:** Margarita Bala, Cristina L Ronchi, Josef Pichl, Vanessa Wild, Stefan Kircher, Bruno Allolio, Stefanie Hahner

**Affiliations:** 1 Department of Medicine I, Endocrine and Diabetes Unit, University Hospital of Wuerzburg, Oberduerrbacherstrasse 6, Wuerzburg D-97080, Germany; 2 Department of Internal Medicine, St. Theresien Hospital of Nuremberg, Nürnberg, Germany; 3 Department of Pathology, University of Wuerzburg, Würzburg, Germany

**Keywords:** Cushing’s syndrome, Kaposi sarcoma, Immunosuppression, Hypercortisolism

## Abstract

**Background:**

Kaposi sarcoma (KS) is a malignant disease most commonly diagnosed in the setting of a human immunodeficiency virus (HIV) infection and in patients receiving immunosuppressive treatment. Pulmonary KS has never been reported in association with endogenous Cushing’s syndrome (CS).

**Case presentation:**

A 60-year-old woman presented with symptoms and signs of CS. Adrenal CS was confirmed by standard biochemical evaluation. Imaging revealed a right adrenal lesion (diameter 3.5 cm) and multiple pulmonary nodules, suggesting a cortisol-secreting adrenal carcinoma with pulmonary metastases. The patient underwent right adrenalectomy with a pathohistological diagnosis of an adrenal adenoma. Subsequent thoracoscopic wedge resection of one lung lesion revealed pulmonary KS with positive immunostaining for human herpes virus 8 (HHV-8). HIV-serology was negative. Hydrocortisone replacement was initiated for secondary adrenal insufficiency after surgery. Post-operative follow up imaging showed complete remission of all KS-related pulmonary nodules solely after resolution of hypercortisolism.

**Conclusion:**

KS may occur in the setting of endogenous CS and may go into remission after cure of hypercortisolism without further specific treatment.

## Background

**Endogenous Cushing’s syndrome** (CS) is a rare disorder with an incidence of ca. 1/100.000 population [1,2]. The symptoms and signs of CS like truncal obesity, moon face, diabetes mellitus, hypertension and muscle weakness result from chronic glucocorticoid excess. Causes of endogenous hypercortisolism include corticotropin-dependent forms (corticotropic adenoma of the pituitary or ectopic corticotropin secretion) and corticotropin independent forms due to adrenal tumors or primary adrenal hyperplasia [1-3]. The diagnosis is based on the clinical presentation, established biochemical tests (consensus paper) and imaging techniques.

**Kaposi sarcoma** (KS) is a vascular, highly malignant tumor involving blood and lymphatic vessels [4] with an incidence of ca. 1.2/100.000 (Surveillance Epidemiology and End Results Program, Cancer statistics Review 2008–2010). This neoplasm is mostly associated with human herpesvirus 8 (HHV-8) infection, a virus also known as Kaposi-sarcoma-associated Herpes virus (KSHV), which may be transmitted sexually, through blood or saliva, or after organ transplantation [5,6]. A dysregulation of the immune system is frequently involved in the pathogenesis of KS [7]. For this reason, KS became more widely known after the onset of the AIDS epidemic, but it has been also described among patients that underwent organ transplantation and subsequent immunosuppressive treatment [5,8]. KS has also been reported after corticosteroid therapy [9]. According to clinical and pathogenetic features, four subtypes of KS are currently distinguished: 1. classic (sporadic or Mediterranean), which mainly affects elderly people, 2. iatrogenic, which is observed in immunosuppressed patients usually after transplantation or after treatment for autoimmune disorders, 3. endemic, in some African countries and 4. epidemic, being human-immunodeficiency-virus (HIV) syndrome-related [10]. The therapy varies according to the type of KS. In the case of epidemic (HIV-related) or iatrogenic KS, treatment primarily aims at immune reconstitution by means of highly active antiretroviral therapy (HAART) or reduction of immunosuppressive therapy, respectively [11,12].

We recently experienced the extraordinary case of an adrenal CS associated with pulmonary KS, which reached a complete remission after treatment of CS by adrenalectomy. To our knowledge this is the first reported case of pulmonary KS related to endogenous CS.

## Case presentation

A 60-year-old woman was admitted to our endocrine department with a diagnosis of Cushing’s syndrome (CS) presumably caused by a cortisol-secreting adrenal carcinoma with pulmonary metastases. The patient reported a progressive course of central obesity, moon-shaped face, muscle atrophy and weakness, asthenia and emotional disturbance over the last six years. During this period she had developed arterial hypertension, which was poorly controlled despite five antihypertensive drugs. Five years ago abdominal magnetic resonance (MR) imaging had been performed in the local hospital for further evaluation of a suspected liver hemangioma, and revealed an incidental finding of a 2.5 cm lesion in the right adrenal. No endocrinological evaluation was performed. Three years before admission she underwent abdominal computed tomography (CT) imaging for evaluation of recurrent hypertensive crises. No change of the adrenal tumor size was found. Screening for pheochromocytoma and primary hyperaldosteronism was negative. In the weeks before presentation at our department the patient complained of polyuria and exertional dyspnoe. The family physician diagnosed diabetes mellitus, which was treated with dietary measures. Furthermore, abdominal CT imaging was repeated, which showed an increase of the adrenal tumor size to 3.3 cm. The patient was subsequently admitted to the local hospital for further endocrinological investigation.

**Figure 1:**
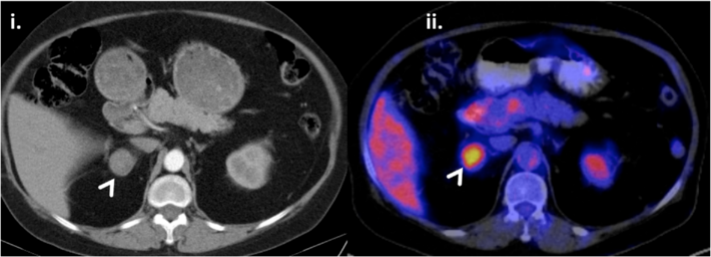
**Imaging of the right adrenal tumor at initial evaluation. i**. CT imaging revealed a 3.3 cm right adrenal tumor (white arrow) **ii**. High uptake of ^18^ F-FDG with standardized uptake value (SUV) of 5.0 for the right adrenal lesion (white arrow).

**Figure 2:**
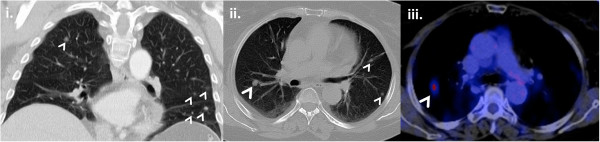
**Imaging of the bilateral pulmonary nodules at initial evaluation. i**. and **ii**. Multiple bilateral pulmonary nodules (white arrows) in CT imaging **iii**. High uptake of ^18^ F-FDG in one pulmonary nodule (white arrow) (SUV of 3.7), highly suspicious for malignancy.

Laboratory tests performed at the local hospital showed failure to suppress cortisol levels after 1 mg dexamethasone and elevated 24-hour urine cortisol secretion. Plasma adrenocorticotropin (ACTH) levels were suppressed indicating an adrenal origin of the hypercortisolism (Table 1). A pulmonary nodule was suspected in the abdominal CT imaging and an additional chest CT revealed multiple bilateral pulmonary nodules which were not described in a CT imaging of the chest performed 5 years earlier. A fluorodeoxyglucose positron emission tomography (FDG-PET) was performed for further evaluation. A high uptake of ^18^ F-FDG for both the adrenal lesion and the pulmonary nodules was observed (Figure 1 and Figure 2).

**Table 1:** Laboratory and endocrine baseline and function tests of the patient before and after adrenalectomy

**Laboratory tests**	**Preoperative values**	**Postoperative values**	**Normal range**
Lymphocyte count (*1000/μl)	1.26	2.89	1-4.05
Lymphocyte (%)	**12.9**	30.6	25-40
Cortisol basal (μg/dl)	**27**	**3.9**	5-25
Cortisol after 1 mg Dexamethason-Suppression-Test (μg/dl)	**18**	**3.5**	< 1.8
Cortisol after short synacthen-Stimulation-Test (μg/dl)	Not applicable	**10.8**	> 18
24-hour urine cortisol (μg/L)	**270**	Not applicable	8-70
Midnight salivary cortisol (μg/dl)	**1.35**	0.09	0-0.15
ACTH (pg/ml)	8.9	6.9	0-46

ACTH = adrenocorticotropic hormone. Bold = altered parameters.

* = multiplied by.

The patient presented in our clinic for further evaluation of the suspected metastatic adrenal carcinoma. The physical examination revealed skin atrophy with bruises, buffalo hump, central obesity and facial hirsutism. Laboratory tests confirmed the presence of ACTH-independent hypercortisolism (Table 1). For histological verification and for “debulking” purposes to potentially reduce the cortisol excess, the patient underwent a right laparoscopic adrenalectomy.

**Figure 3:**
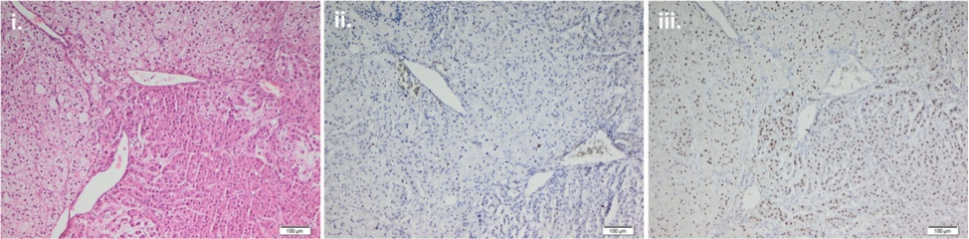
**Paraffin sections with immunohistochemical staining of the cortisol-secreting adrenal lesion. i**. H-E staining **ii**. Negative staining of Ki67 **iii**. Positive nuclear staining of SF1.

**Figure 4:**
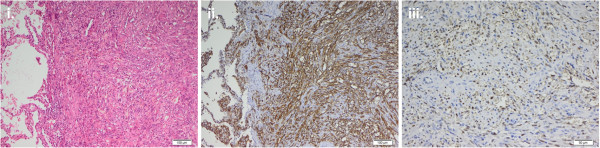
**Paraffin sections with immunohistochemical staining of the left lobe lung biopsy. i**. H-E staining **ii**. Positive membranous and cytoplasmic staining of CD31 **iii**. Positive nuclear staining of human herpes virus 8 (HHV-8).

Unexpectedly, the histological report indicated an adrenocortical adenoma with no evidence of malignancy. The tumor size was 3.5 cm and the Weiss score was 0, compatible with a definitive diagnosis of a **benign cortisol-secreting adrenocortical tumor** (Figure 3). Postoperatively signs of CS gradually receded and laboratory testing revealed secondary adrenal insufficiency (Table 1). The patient received intra- and postoperative glucocorticoid coverage followed by chronic replacement therapy for adrenal insufficiency. A thoracoscopic wedge resection with biopsies of the left upper and lower lobe was performed. Histopathology showed spindle-shaped cells with positive immunostaining for CD31, CD34 and for human herpes virus 8 (HHV-8) (Figure 4), consistent with a diagnosis of **pulmonary Kaposi’s sarcoma**. Neither skin lesions nor involvement of the gastrointestinal tract or other organs were detected. Gastroscopy and colonoscopy were unremarkable. Human immunodeficiency virus (HIV) testing was negative and testing of the immune status showed normal CD4 cell counts (1014 cells/mm^3^; normal range: 410–1590 cells/mm^3^) as well as a normal CD4/CD8 ratio (1.18; normal range: 0.8-4.2) eight weeks after adrenalectomy. The differential white blood cell count revealed relative lymphopenia before the adrenalectomy which was normalised 8 weeks after adrenalectomy (Table 1).

**Figure 5:**
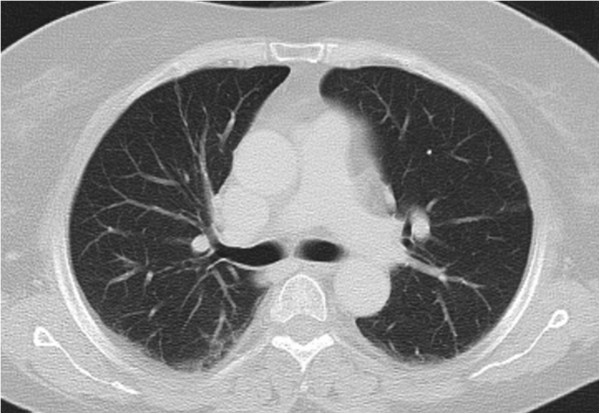
Restaging with CT three months after the adrenalectomy showed disappearance of all KS-induced pulmonary nodules.

Palliative treatment with systemic combination chemotherapy was considered after the diagnosis of advanced KS was established, but was refused by the patient. The patient experienced significant clinical improvement of her hypercortisolism-related clinical symptoms such as normalization of hypertension under antihypertensive medication, fat redistribution and gradual recovery of muscular strength. Restaging was performed with chest and abdominal CT three months (Figure 5) and with FDG-PET/CT six months after the adrenalectomy and showed complete disappearance of all KS-induced pulmonary nodules.

## Discussion

We here describe a case of adrenal CS associated with pulmonary KS with complete remission after successful treatment of CS by unilateral adrenalectomy. It is well known that cortisol excess causes a state of immunosuppression [13,14]. KS occurs in patients with an immunocompromised state mainly due to advanced HIV disease or in transplant patients [4,8]. The etiologic agent of almost all forms of KS is HHV-8, but additional immunosuppression is necessary for the manifestation of this malignant disease [5]. Corticosteroids seem to play a role in the development of KS. Classical KS risk was found to be strongly and independently associated with oral corticosteroid use [15]. In addition, KS has also been reported in association with the immunosuppressive effects of systemic steroids in various clinical conditions (iatrogenic KS). After withdrawal or reduction of corticosteroids a partial or complete regression of KS may occur [16-19]. The exact pathophysiological mechanism of this association is not yet understood. The increased incidence and severity of KS in transplant recipients was not found to be associated with direct cellular effects of corticosteroids on HHV8 reactivation [20]. Glucocorticoids seem however to induce KS cell proliferation through the regulation of transforming growth factor-β [21]. Furthermore, in AIDS-KS tumor biopsies high expression of glucocorticoid receptors (GRs) was detected as well as an up-regulation of GRs by KS-growth-promoting factors like tumor necrosis factor-α [22].

A literature search revealed two cases of endogenous ACTH dependent CS which both were associated with cutaneous KS [23,24]. In the first case a 43-year-old HIV-negative woman from Turkey developed CS due an ectopic hypothalamic adrenocorticotropic hormone-secreting adenoma. The patient developed diffuse skin lesions on her abdomen, legs, eyelids, and toes, which were proven to be KS by a skin biopsy. Within one month after transcranial surgery and cure of CS, all skin lesions disappeared spontaneously [23]. In the second case a 54-year-old Hispanic HIV-negative man developed CS due to a pituitary ACTH-secreting adenoma. Coincidentally he showed numerous raised, purplish, non-blanching plaques extended throughout his lower extremities, which were also proven to be KS by skin biopsy. He underwent repeated transphenoidal surgeries followed by bilateral adrenalectomy. The patient received additional treatment with systemic combination chemotherapy with liposomal doxorubicin and paclitaxel resulting in a significant decrease of the KS lesions [24].

In our case the patient had adrenal CS and presented at a late stage of KS (pulmonary Kaposi) indicating longer term severe immunosuppression. Clinical symptoms of CS were present for at least 5 years and an incidental adrenal lesion had already been diagnosed in MR imaging 5 years before. CS is associated with immune dysregulation and CS patients have been shown to display impairment in immune regulation, including changes in both the percentage of specific lymphocyte subsets and specific lymphocyte functional activities [25]. The patient presented in our clinic for further evaluation of suspected metastatic adrenal carcinoma, therefore we did not initially perform any specific preoperative examination of the patient’s immune system. After establishment of the diagnosis of Kaposi Sarcoma, specific testing of the immune status showed normal CD4 cell counts and normal CD4/CD8 ratio. However, testing had been performed eight weeks after adrenalectomy and after resolution of the hypercortisolism. Treatment of CS resulted in a complete remission of KS despite a visceral manifestation which is usually associated with a poor prognosis [26]. This demonstrates a key role of cortisol excess in the pathogenesis and the high relevance of restoration of immune function for treatment of KS.

Our case also emphasizes the importance of hormonal workup in adrenal incidentalomas including screening for cortisol-producing adenoma, hyperaldosteronism and pheochromocytoma which was delayed and only incompletely performed in our patient.

The combination of cortisol excess with positive FDG-uptake of both the adrenal tumor and the pulmonary nodules clearly suggested a cortisol-secreting adrenal carcinoma with pulmonary metastases. Malignant adrenal lesions have a higher standardized uptake value (SUV) than benign lesions, but in most cases have a tumor diameter >5 cm [27]. Moreover, a high SUV is occasionally also found in benign hormone secreting adenomas [27]. Furthermore, the long standing history of an adrenal incidentaloma in our patient also pointed to the possibility of a benign tumor, although some increase in diameter was documented prior admission. These aspects supported the indication for a removal of the adrenal tumor which otherwise would have remained a matter of debate in stage IV ACC [28].

Currently, four different types of KS are distinguished, the classic, endemic, epidemic HIV related and the iatrogenic type which is related to treatment with immunosuppressants. Iatrogenic KS shows similar features as have been observed in the patients with endogenous CS. Based on our case and the two earlier reports of cutaneous KS in ACTH dependent CS, we suggest adding a fifth type of KS with endogenous corticosteroid-excess as cause of KS (Table 2) being, however, closely related to the iatrogenic phenotype. The association of KS with both exogenous and endogenous glucocorticoid excess requires a concurrent HHV-8 infection. Similar to KS in association with iatrogenic immunosuppression, which may resolve completely after reduction or discontinuation of immunotherapy, effective treatment of endogenous hypercortisolism may also successfully lead to remission of KS.

**Table 2:** Clinical types of Kaposi sarcoma (KS)

**Type of KS**	**Cutaneous manifestation**	**Visceral manifestation**	**Clinical course**
Classic (sporadic or Mediterranean)	Yes (often limited to lower extremity)	No (uncommon)	Low malignant potential
Endemic (African)	Yes	Yes	Indolent, occasionally aggressive progression
Epidemic AIDS-associated	Yes	Yes	Indolent, occasionally aggressive progression. May regress with HIV treatment
Latrogenic (immunosuppression-related)	Yes	Yes	May heal spontaneously with reduction or discontinuation of immunosuppression. May be aggressive.
**Endogenic (endogenous corticosteroid-excess-related)**	**Yes (in the literature)**	**Yes (in our case)**	**May regress after treatment of hypercortisolism**

Endogenic (endogenous corticosteroid-excess-associated) KS represents a potential new form of Kaposi sarcoma.

In bold is underlined the new type of Kaposi sarcoma.

## Conclusions

Our findings demonstrate that endogenous immunosuppression due to Cushing’s syndrome may lead to pulmonary Kaposi sarcoma which may regress after treatment of hypercortisolism. The data emphasize the key role of glucocorticoid excess in the pathogenesis of Kaposi Sarcoma and the high relevance of restoration of immune function for its treatment. The data further emphasize the need of careful evaluation of patients with adrenal lesions.

### Consent

Written informed consent was obtained from the patient for publication of this Case report and any accompanying images. A copy of the written consent is available for review by the Editor of this journal.

## Competing interests

The authors declare that they have no competing interests.

## Authors’ contributions

MB, CLR and SH treated the patient and drafted the article. JP initially treated the patient. VW and SK carried out the immunoassays. BA critically revised the manuscript. All authors read and approved the final manuscript.

## Pre-publication history

The pre-publication history for this paper can be accessed here:

http://www.biomedcentral.com/1472-6823/14/63/prepub
